# Post-herniorrhaphy extubation technique in pediatric patient with congenital diaphragmatic hernia and VACTERL association: A case report

**DOI:** 10.1016/j.amsu.2021.102801

**Published:** 2021-09-09

**Authors:** Diajeng Putri Iracily, Desy Rusmawatiningtyas, Firdian Makrufardi, Intan Fatah Kumara, Nurnaningsih, Edy Moeljono

**Affiliations:** Department of Child Health, Faculty of Medicine, Public Health and Nursing, Universitas Gadjah Mada/Dr. Sardjito Hospital, Yogyakarta, 55281, Indonesia

**Keywords:** Extubation technique, Congenital diaphragmatic hernia, VACTERL association, Pediatric, Case report

## Abstract

**Introduction:**

and importance: Congenital diaphragmatic hernia (CDH) is a condition characterized by a defect in the diaphragm causing protrusion of abdominal organs into the thoracic cavity. Comprehensive management, definitive surgical procedures and postoperative care are able to significantly reduce morbidity and mortality in post-herniorrhaphy patients. Here, we reported a case of post herniorrhaphy pediatric patient with a challenge in extubation.

**Case presentation:**

A 4-month-old girl with a chief complaint of respiratory distress from was admitted to our Pediatric Intensive Care Unit. The diagnosis of diaphragmatic hernia was confirmed through small bowel follow-through radiological examination. Definitive treatment of laparotomy and herniorrhaphy were then done. The special technique for extubation was applied. Currently, the patient survives without any sequelae awaiting stoma closure.

**Clinical discussion:**

Extubation is the removal of an endotracheal tube when it is no longer needed. In mechanically ventilated patients, extubation can be performed in two ways, either using the tracheal suction catheter (TSC) or positive pressure breath (PPB) techniques. Studies show that the PBB extubation technique has better patient outcomes compared to the TSC technique. However, the TSC technique is more commonly done by medical professionals. We used the PPB technique because there were recurrent atelectases in the left lung.

**Conclusion:**

This case report illustrates extubation technique in a post-herniorrhaphy patient with congenital diaphragmatic hernia and VACTERL association. Moreover, several options of extubation techniques can be used for extubation procedure in pediatric patient with CDH.

## Highlights

• Most children with congenital diaphragmatic hernia comes with respiratory problems.
• Comprehensive management, definitive surgical procedures and postoperative care.
• Good outcome of comprehensive management and appropriate extubation technique.

## 1 Introduction

Congenital Diaphragmatic Hernia (CDH) is a condition characterized by a defect in the diaphragm that causes the protrusion of abdominal organ into the chest cavity, interfering with the normal development of the lungs. According to the literature, the incidence of CDH ranges from 0.8 to 5 out of 10,000 live births [1]. Bochdalek initially explained the concept of herniation due to failure of pleuroperitoneal canal closure, while Morgagni described hernias which occur due to failure of anterior pleuroperitoneal membrane fusion with the sternum and costal cartilage during embryogenesis [2]. Postero-lateral hernias (Bochdalek hernias) are the most common type of hernia (70–75%) with the majority occurring on the left side (85%) and less common on the right side (13%) or bilaterally (2%). Anterior hernia (Morgagni Hernia) (23–28%) and central hernia (2–7%) are other types of hernia. The diaphragm starts to develop at 4 weeks of gestation and is fully formed at 12 weeks of gestation. Defects can vary from a small gap of the posterior margin of the muscle to the absence of a diaphragm [1].

Most postoperative patients with diaphragmatic hernia require ventilatory support and have difficulty weaning or extubation [3]. Extubation can be done with two techniques in mechanically ventilated patients, either with a tracheal suction catheter (TSC) or with a positive pressure breath (PPB) [4]. While studies have indicated that the PBB extubation approach has a better patient result than the TSC technique, the TSC technique is used by medical practitioners more frequently [6]. Here, we reported a case of pediatric patient with post-herniorrhaphy CDH with a challenge in extubation. Furthermore, this case report explains several options of extubation techniques that can be used for extubation procedure in pediatric patient with CDH. This case was reported in line with the Surgical CAse REport (SCARE) criteria [7].

## 2 Case presentation

A 4-month-old girl was referred from a private hospital in Yogyakarta with respiratory distress. The patient was born in a private hospital by cesarean section due to pre-partum bleeding. The patient cried immediately after birth with a birth weight of 2800 g. After birth, congenital anomaly were found, which is congenital talipes equinovarus (CTEV) and anorectal malformation with rectovaginal fistula. The patient underwent fistula closure surgery and colostomy at 2 different times. The procedure was carried out at the secondary hospital before the patient being referred to our hospital. Prior to the colostomy procedure, a radiological examination of a chest X-ray (pre-operative screening) showed there was no diaphragmatic hernia.

**Fig. 1:**
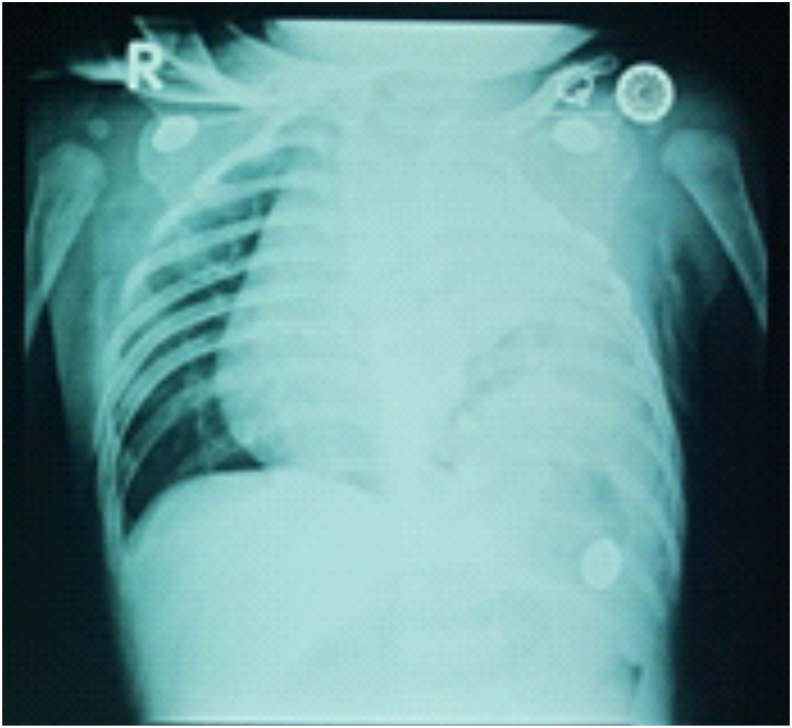
Chest X-ray at the age of 4 months showed intestines in the left thoracic cavity, leading to a diaphragmatic hernia.

**Fig. 2:**
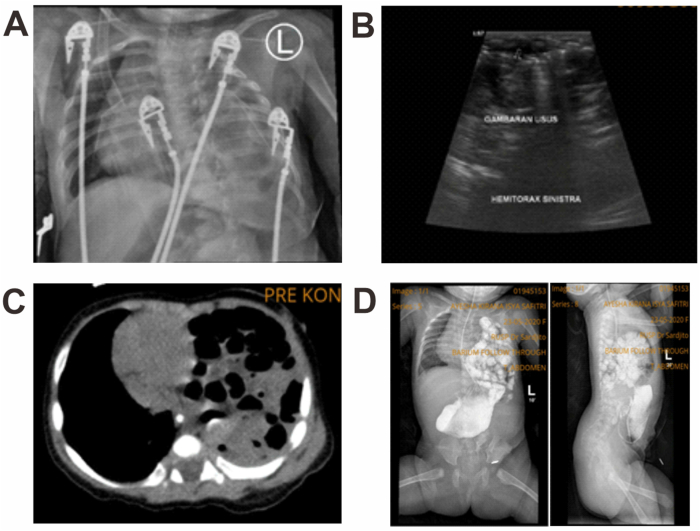
A. Chest X-ray at the age of 4 months showed a lucent lesion with septation in the left thoracic cavity; B) Thoracic ultrasound showed the left thoracic cavity filled with intestines; C) CT scan of the thorax showed elevated small intestine and colon which pushed the mediastinum to the contralateral (right) side; and D) X-ray of small bowel follow-through (AP and lateral position) showed the intestines in the left thoracic cavity.

At the age of 4 months, the patient was planned for posterior sagittal anorectoplasty (PSARP) surgery. The patient underwent a preoperative radiological examination in the form of a chest X-ray and the result showed there was no diaphragmatic hernia, however the left diaphragm was higher than usual. During hospitalization prior to the PSARP procedure, the patient developed respiratory distress suspected to be due to pneumonia. The patient was evaluated using chest X-ray and the result showed diaphragmatic hernia. Moreover, the expertise from radiology department concluded that this was atelectasis and the pulmonary hypoplasia was not proven. We also excluded the possibility of pulmonary hypoplasia since the patient had no abnormality on 20-days-old X-ray (Fig. 1). The two X-rays were performed within a day apart. The patient was then referred to our hospital for further treatment. Because there was a significant difference from the previous chest X-ray in just 1 day, other diagnostic examinations were done including chest X-ray (Fig. 2A), thorax ultrasound (Fig. 2B), thorax CT scan (Fig. 2C) and barium follow-through (Fig. 2D). Since the results of all supporting examinations were conclusive for diaphragmatic hernia, it was decided that the patient would undergo laparotomy and herniorrhaphy. The blood gas analysis showed pH: 7.38 (Reference: 7.35–7.45), pCO2: 47 mmHg (Reference: 35–45), pO2: 124.1 mmHg (Reference: 80–95), HCO3 28 mmol/l (Reference: 22–26) and base excess: 2.7. We performed the echocardiography and found patent foramen ovale, structurally and functionally normal heart, and no pericardial effusion. We did not install any ECMO due to the unavailability of the tool in our low-resource PICU. During surgery, a 2 cm hole was found in the left diaphragm, indicating a Bochdalek-type diaphragmatic hernia. There was no history of trauma. The post-operative chest X-ray examination showed that the diaphragmatic hernia was no longer visible.

Three days after surgery, there was difficulty to extubate the patient from the ventilator. The patient experienced desaturation each time the ventilator machine's setting was reduced, and subsequently, developed pneumonia, severe sepsis, septic shock, and disseminated intravascular coagulation (DIC).Twelve days after surgery, all complications during post-operative care had been resolved and the ventilator reached minimal settings. The patient was extubated and oxygenation was done through a non-invasive ventilator. After extubation, the stridor improved with the administration of intravenous nebulized adrenaline and methylprednisolone. With the findings described above, the patient was considered to have laryngeal edema and treated accordingly. Two days after extubation, there was recurrent respiratory distress with no breath sounds in the left lung. From the chest X-ray evaluation, there was pneumonia in the right lung, apico-posterior segment atelectasis of the left lung and prominent thymus. The thorax ultrasound showed atelectasis of the superior lobe of the left lung. The patient was managed for pneumonia in the right lung and recurrent atelectasis. She was again intubated and given antibiotics as a treatment for pneumonia. The results of the post-re-intubation chest X-ray (on the same day) showed pneumonia in the right lung, prominent thymus and normal heart. There were no signs of left pulmonary atelectasis at that time.

During treatment, the patient failed extubation twice. Total ventilator days reached 25 days. We tried to exclude other factors to explain difficulty in extubation including infection, pulmonary hypertension, and pulmonary hypoplasia. Ventilator-associated pneumonia (VAP) in this patient proven from the tracheal aspirate that showed *Acinetobacter baumannii*. We gave complete course of antibiotics for VAP. Pulmonary hypertension was not proven from serial echocardiography results. Pulmonary hypoplasia was also not proven from serial x-ray before and after the surgery. Stage I hypertension were under control using amlodipine and spironolactone. After completing antibiotic therapy for pneumonia, extubation was performed using a special method. We used the PPB technique. The endo-tracheal tube (ETT) was removed following positive pressure ventilation with 100% oxygen with a re-breathing bag. In PPB, the ETT is withdrawn at the end of inspiration when the lungs are fully inflated, then a cough response occurs, clearing the airway from mucous secretions and preventing recurrent lung atelectasis.

The patient was successfully discharged with an improved condition and was active with good awareness and no recurrent respiratory distress. Defecation still occurred through the stoma and she still needs to undergo the procedure to close the stoma. Fluid and nutritional needs are fulfilled through a nasogastric tube. Currently, the patient can live normally without any sequelae.

**Fig. 3:**
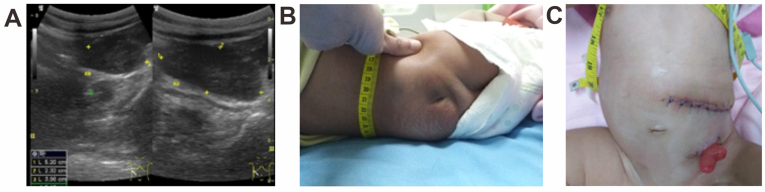
A) Abdominal ultrasound showed no abnormalities in the right kidney, but the left kidney was not visualized; B) Sacral dimple; and C) Stoma on the abdominal wall post-colostomy at 3 weeks of age (this image was taken after diaphragmatic hernia surgery).

The patient's final diagnosis was VACTERL association with the four following findings: left renal agenesis (Fig. 3A), sacral dimple (suspected spina bifida) (Fig. 3B), post-colostomy anorectal malformation (Fig. 3C) and congenital talipes equinovarus (CTEV). There were no cardiac abnormalities as shown from the echocardiography examinations and also stage I hypertension occurs most likely due to renovascular disease. The patient was consulted to the pediatric nephrology department and received amlodipine and spironoloactone.

## 3 Discussion

Here, we reported a case of 4-month-old female patient with vertebral defects, anal atresia, cardiac defects, tracheo-esophageal fistula, renal anomalies, and limb abnormalities (VACTERL) association who was referred to our hospital due to respiratory distress caused by the CDH. At a mean gestational age of 24 weeks, prenatal ultrasonography can detect more than 50% of CDH patients [19]. But due to low socioeconomic status, the patient's mother never had the fetal ultrasound, which is very common in Indonesia setting.

Approximately 5–45.5% of CDH may appear healthy during the newborn period, but abnormality can manifest in later life [20]. We assessed this patient as congenital diaphragmatic hernia due to the absence of all types of trauma on the thorax and abdominal region before the episode of protrusion of abdominal contents into the thoracic cavity. Thus, we did not find any sign of trauma from the physical examination. The defect of the diaphragm occurs congenital but the abdominal contents did not pass into thoracic cavity during the newborn period due to the small size of the hole. Incidentally, this patient was crying out load at the time of intravenous line insertion (preparation of posterior sagittal anorectoplasty surgery at the previous hospital), increasing the intraabdominal pressure, leads to protrusion of abdominal contents due to thoracic cavity although the size of the hole was small. After the surgery, we found 2 cm left side bochdalek type diaphragmatic hernia. Highly connected to the data from the literature, bochdalek (posterolateral) hernias comprise approximately 80%–90% of all CDH. About 85% of Bochdalek hernias occur on the left side, about 10% on the right and approximately 5% are bilateral [21].

Children with CDH most often present with respiratory problems [8]. Defects in the diaphragm cause the abdominal viscera to herniate into the thoracic cavity resulting in abnormal lung development [5]. The main pathophysiology underlying CDH appears to be a combination of pulmonary immaturity and hypoplasia leading to pulmonary hypertension. This condition can be exacerbated by right ventricular hypertrophy resulting in ventricular dysfunction. Pulmonary hypoplasia occurs on the ipsilateral side of the herniation, while the contralateral side is affected to varying degrees. Hypoplasia was originally thought to be the result of physical compression of the lungs by the abdominal organs which inhibits lung development [9].

Surgery to repair the diaphragmatic defect consists of a subcostal incision with removal of the abdominal organs from the thorax and complete closure of the defect. Currently, the use of minimally invasive surgical techniques to repair CDH is becoming more common, but involves a significantly higher risk of recurrence [5]. This patient received open surgical management as definitive therapy. Abdominal organs were removed from the chest cavity and the damage was permanently closed.

After the surgery, patients require ventilatory support. Sepsis has a significant impact on the duration of ventilator use and the length of stay in the pediatric intensive care unit (PICU). Studies show that patients hospitalized for CDH have a high incidence of sepsis, mostly due to ventilator-associated pneumonia (VAP).

Extubation is the removal of an ETT when it is no longer needed [10]. This procedure can lead to complications such as bronchospasm which can result in desaturation [11,12]. Clinical intolerance and signs of weaning failure include a systolic blood pressure of more than 180 mmHg or a pressure change of more than 20%. Antihypertensive therapy should be given to the patient [22]. In this case, the patient's condition was controlled using amlodipine and spironolactone. During use of a mechanical ventilator, mucosal secretions accumulate in the subglottic space, above the ETT cuff. There is a possibility for the secretions to be aspirated when the ETT cuff is deflated and extubated. In some cases, this complication can lead to failure of extubation [13]. In mechanically ventilated patients, extubation can be performed in two ways, either using the tracheal suction catheter (TSC) or the positive pressure breath (PPB) techniques [17]. In the TSC technique, the ETT is first aspirated and then along with the suction catheter, it is removed. In this case, the technique was done by two experienced medical professionals. For the PPB technique, the ETT is withdrawn after giving PPB using 100% oxygen with a re-breathing bag. This technique can be performed by only one medical professional. In the TSC technique, although mucosal secretions from the chest or pharynx are removed using suction, the catheter may not be able to remove all mucosal secretions. In addition, the negative pressure generated by the suction catheter facilitates leakage of mucosal secretions into the distal airways which can lead to pulmonary aspiration and atelectasis [18]. In the PPB technique, the ETT is expelled at the end of inspiration when the lungs are fully inflated, causing a cough reflex that can clear the airway from all secretions and prevent pulmonary atelectasis [6].

Studies have shown that the PBB extubation technique has better patient outcomes compared to the TSC technique, however, TSC technique is more commonly done by medical professionals [9]. In this patient, we used the PPB technique because there were recurrent atelectases in the left lung. The recurrent atelectases in this patient may be due to either pneumonia or inadequate extubation techniques. VAP is a major infection threat in children with CDH [6]. We administered additional antibiotics and then we extubated this patient with the PPB technique. This procedure showed good results. There was no recurrence of atelectasis after the usage of the PPB technique and antibiotics. In previous extubations, we have not used this technique. The literature also showed similar results, and the outcome of extubation with the PPB technique proved to be more superior in terms of incidence of complications. The incidence of post-extubation pneumonia was lower in the PPB group since positive pressure could reduce the leakage of colonized subglottic mucosal secretions into the distal airway. Hence, one study considers that the technique should be used for everyday practice in the intensive care units [16]. Furthermore, the appropriate extubation technique will provide good outcome and no recurrent respiratory distress in congenital anomaly patients [9].

In general, the survival rate of children with CDH varies due to differences in treatments in various facilities, including the availability of ventilators, extracorporeal membrane oxygenation (ECMO), surfactant and surgical time. In recent years, the survival rate of patients with CDH has increased significantly between 50% and 90% but the condition is followed with long-term morbidity [14]. Children with CDH require long-term and multidisciplinary monitoring including standardized developmental examinations, especially in high-risk children (Example: Children who require respiratory support for more than 30 days or children on ECMO) [15].

## 4 Conclusions

This case report illustrates the good outcome of comprehensive management and appropriate extubation technique in patient with congenital diaphragmatic hernia and VACTERL association. Moreover, several options of extubation techniques can be used for extubation procedure in pediatric patient with CDH.

## Ethical approval

The informed consent form was declared that patient data or samples will be used for educational or research purposes. Our institutional review board also do not provide an ethical approval in the form of case report.

## Funding

The authors declare that this study had no funding source.

## Author contribution

Diajeng Putri Iracily, Desy Rusmawatiningtyas, Nurnaningsih, Edy Moeljono conceived the study and approved the final draft. Diajeng Putri Iracily, Desy Rusmawatiningtyas, Firdian Makrufardi, Intan Fatah Kumara and Nurnaningsih drafted the manuscript, and critically revised the manuscript for important intellectual content. Diajeng Putri Iracily, Desy Rusmawatiningtyas, Firdian Makrufardi, Intan Fatah Kumara, Nurnaningsih and Edy Moeljono facilitated all project-related tasks.

## Registration of research studies

This is not a ‘first in humans’ report, so it is not in need of registration.

## Guarantor

Desy Rusmawatiningtyas.

## Consent

Written informed consent was obtained from the patient for publication of this case report and accompanying images. A copy of the written consent forms is available for review by the Editor-in-Chief of this journal on request.

## Funding source

This research did not receive any specific grant from funding agencies in the public, commercial, or not-for-profit sectors.

## Provenance and peer review

Not commissioned, externally peer-reviewed.

## Declaration of competing interest

No potential conflict of interest relevant to this article was reported.
